# Clade C HIV-1 isolates circulating in Southern Africa exhibit a greater frequency of dicysteine motif-containing Tat variants than those in Southeast Asia and cause increased neurovirulence

**DOI:** 10.1186/1742-4690-10-61

**Published:** 2013-06-08

**Authors:** Vasudev R Rao, Ujjwal Neogi, Joshua S Talboom, Ligia Padilla, Mustafizur Rahman, Cari Fritz-French, Sandra Gonzalez-Ramirez, Anjali Verma, Charles Wood, Ruth M Ruprecht, Udaykumar Ranga, Tasnim Azim, John Joska, Eliseo Eugenin, Anita Shet, Heather Bimonte-Nelson, William R Tyor, Vinayaka R Prasad

**Affiliations:** 1 Department of Microbiology and Immunology, Albert Einstein College of Medicine, 1300 Morris Park Avenue, Bronx, NY, 10461, USA; 2 Division of Clinical Virology, Department of Microbiology and Pediatrics, St. John’s Medical College and Hospital, Sarjapur Road, Bangalore, India; 3 Department of Psychology, Arizona State University, Tempe, AZ, USA; 4 Arizona Alzheimer’s Consortium, Phoenix, AZ, USA; 5 Atlanta VA Medical Center, Decatur, GA, USA; 6 International Centre for Diarrhoeal Disease Research, Mohakhali, Dhaka, Bangladesh; 7 Immunology and Molecular Pathology Program Emory University, Graduate Division of Biological and Biomedical Sciences, Atlanta, GA, USA; 8 Nebraska Center for Virology and the School of Biological Sciences, University of Nebraska-Lincoln, Lincoln, NE, USA; 9 Dana-Farber Cancer Institute and Harvard Medical School, Boston, MA, USA; 10 Jawaharlal Nehru Centre for Advanced Scientific Research, Bangalore, India; 11 Department of Psychiatry and Mental Health, University of Cape Town, Cape Town, South Africa; 12 Public Health Research Institute (PHRI), Newark, NJ, USA; 13 Department of Microbiology and Molecular Genetics, UMDNJ, Newark, NJ, USA; 14 Department of Neurology, Emory University School of Medicine, Atlanta, GA, USA

**Keywords:** Clade C HIV-1, Subtype C HIV-1, HIV-1 Tat, Tat dicysteine, Neuropathogenesis, SCID-HIVE mouse model, HIV-1 Tat C31S polymorphism, Neuroaids, HIV dementia

## Abstract

**Background:**

HIV-1 Clade C (Subtype C; HIV-1C) is responsible for greater than 50% of infections worldwide. Unlike clade B HIV-1 (Subtype B; HIV-1B), which is known to cause HIV associated dementia (HAD) in approximately 15% to 30% of the infected individuals, HIV-1C has been linked with lower prevalence of HAD (0 to 6%) in India and Ethiopia. However, recent studies report a higher prevalence of HAD in South Africa, Zambia and Botswana, where HIV-1C infections predominate. Therefore, we examined whether Southern African HIV-1C is genetically distinct and investigated its neurovirulence. HIV-1 Tat protein is a viral determinant of neurocognitive dysfunction. Therefore, we focused our study on the variations seen in *tat* gene and its contribution to HIV associated neuropathogenesis.

**Results:**

A phylogenetic analysis of *tat* sequences of Southern African (South Africa and Zambia) HIV isolates with those from the geographically distant Southeast Asian (India and Bangladesh) isolates revealed that Southern African *tat* sequences are distinct from Southeast Asian isolates. The proportion of HIV − 1C variants with an intact dicysteine motif in Tat protein (C30C31) was significantly higher in the Southern African countries compared to Southeast Asia and broadly paralleled the high incidence of HAD in these countries. Neuropathogenic potential of a Southern African HIV-1C isolate (from Zambia; HIV-1C_1084i_), a HIV-1C isolate (HIV-1_IndieC1_) from Southeast Asia and a HIV-1B isolate (HIV-1_ADA_) from the US were tested using *in vitro* assays to measure neurovirulence and a SCID mouse HIV encephalitis model to measure cognitive deficits. *In vitro* assays revealed that the Southern African isolate, HIV-1C_1084i_ exhibited increased monocyte chemotaxis and greater neurotoxicity compared to Southeast Asian HIV-1C. In neurocognitive tests, SCID mice injected with MDM infected with Southern African HIV-1C_1084i_ showed greater cognitive dysfunction similar to HIV-1B but much higher than those exposed to Southeast Asian HIV − 1C.

**Conclusions:**

We report here, for the first time, that HIV-1C from Southern African countries is genetically distinct from Southeast Asian HIV-1C and that it exhibits a high frequency of variants with dicysteine motif in a key neurotoxic HIV protein, Tat. Our results indicate that Tat dicysteine motif determines neurovirulence. If confirmed in population studies, it may be possible to predict neurocognitive outcomes of individuals infected with HIV-1C by genotyping Tat.

## Background

Human immunodeficiency virus type 1 (HIV-1) associated neurocognitive disorders (HAND) comprise a spectrum of conditions that range from asymptomatic neurocognitive impairment (ANI) and mild neurocognitive disorder (MND) to the most severe form, HIV-associated dementia (HAD) [1]. Distinct clades of HIV-1 or their recombinants dominate a given geographic region. HIV − 1C, the predominant clade prevalent in Africa and Southeast Asia, is responsible for greater than 50% of all new infections worldwide [2]. Studies have reported a lower prevalence (0%-6%) of HAD in countries where HIV-1C is the dominant clade (e.g., India, Ethiopia) [3,4]. This is in contrast to studies in geographic regions (E.g., United States) that show higher prevalence of HAD (15 - 30%) where HIV-1B is prevalent [5,6].

HIV-1 Tat protein is one of the key viral determinants of neurocognitive disorders [7] Infiltration of mononuclear phagocytes in brain tissue is a hallmark of HAD. Tat, in addition to its key role in LTR-transactivation during viral replication, plays a pivotal role in monocyte chemotaxis and neurotoxicity. Monocyte chemotaxis driven by Tat is mediated by both direct and indirect processes. Direct chemotaxis has been demonstrated *in vitro* using recombinant Tat protein and this activity of Tat has been mapped to the C30C31 dicysteine motif in Tat [8,9]. Such a dicysteine motif is also a key feature of β-chemokines such as CCL2, MIP1α and RANTES [8]. Tat has also been shown to induce the secretion of CCL2 from HIV-infected macrophages thus amplifying the chemokine gradient which help recruit additional mononuclear phagocytes across the blood–brain barrier into the brain [10,11]. This property of Tat is based on its homology to CCL2 and its ability to bind to CCR2 which triggers the induction of CCL2 by macrophages exposed to Tat or HIV-infected cells [12]. The infiltration of HIV − infected macrophages in the brain leads to the secretion of viral proteins such as Tat and gp120, both of which have been shown to be neurotoxic [13,14]. In vivo, the presence of HIV proteins is known to result in the loss of integrity of neuronal dendritic arbor in frontal lobe and hippocampus and this is associated with neuronal apoptosis [7,15-17].

We previously showed that in ~90% of clade C HIV isolates, *tat* gene displays a C31S polymorphism and is defective for monocyte chemotaxis [18]. *In vitro* Boyden chamber assays using HIV-infected monocyte-derived macrophage (MDM) supernatants showed that monocyte recruitment by the Indian clade C HIV-1C_IndieC1_-infected medium was significantly lower than that by the US clade B HIV-1B_ADA_[19]. Depletion of Tat protein from the HIV-infected MDM supernatants by immune-adsorbtion abrogated the differential monocyte recruitment by the two clades of HIV-1. Based on these findings, we had proposed that the chemotaxis defect associated with Tat C31S polymorphism is responsible for the lower prevalence of HAD in India [18]. We used a severe combined immunodeficiency (SCID) HIV Encephalitis (HIVE) mouse model in which intracranial injection of clade B HIV-infected MDMs leads to neuropathology and neurocognitive defects that parallel those found in HAD patients [20,21]. In this model, clade C HIV_IndieC1_ isolate caused milder neuropathology and reduced neurocognitive deficits compared with clade B isolate (HIV-1B_ADA_) [19]. To understand the mechanism underlying clade differences between B and C Tat proteins, Campbell et al. examined Tat-binding to CCR2, intracellular calcium release in response to CCR2-Tat interaction, monocyte migration and the induction of TNF-α [22]. They showed that while clade B Tat specifically binds CCR2, induces robust levels of intracellular calcium influx and TNF-α and causes monocyte migration, clade C Tat (with a C31S substitution) was defective for these functions. In addition to the induction of CCL2, it is known that HIV-1 Tat induces several proinflammatory cytokines. Several groups have shown that *in vitro* purified recombinant HIV-1B Tat induces monocytes to produce proinflammatory cytokines to a greater extent than does HIV-1C Tat [22-25].

The mechanism of neurotoxicity of HIV-1 Tat protein is N-methyl-d-aspartic acid (NMDA) receptor-dependent. Two main mechanisms of NMDA receptor activation by HIV-Tat have been described. In one, an interaction of HIV-Tat with surface receptors, such as low-density lipoprotein receptor-related protein (LRP) and formation of a macromolecular complex leads to neuronal apoptosis [26]. Second, a direct binding of HIV-Tat protein to the (LRP) NMDAR has been shown to cause neuronal killing [27]. Furthermore, it has been shown that NMDA receptor activation is clade dependent in that TatB protein bound to NMDA receptor, while TatC did not [28].

In sharp contrast to reports from India that showed low prevalence of HAD, recent studies from three countries in Southern Africa where HIV-1C is the predominant clade have reported a much higher prevalence of HAD or HAND. South Africa and Botswana have reported a 25% and 38% incidence of HAD respectively [29-32], while Zambia has reported a 22% incidence of HAND [32]. Southern Africa and Southeast Asia are the two main geographic regions where HIV-1C is prevalent. We hypothesized that the Southern African HIV-1C isolates are genetically distinct from those circulating in Southeast Asia with respect to their neuropathogenic potential. Based on previous findings that have implicated *tat* polymorphism as being responsible for clade differences in the incidence of HAD, we characterized HIV-1 Tat exon 1 from both geographical regions. We analyzed not only the sequences available in the databases from countries where HIV-1C is prevalent, but also the sequences derived from newly recruited patients in selected countries. We report here that *tat* sequences from Southern African HIV-1 isolates are genetically distinct from those in Southeast Asian countries. We also found that a greater proportion of Southern African HIV − 1C isolates examined (11% to 26%) encoded dicysteine motif C30C31, whereas much lower proportions (1-3%) of HIV-1C from Southeast Asia encoded this motif.

To determine if the higher incidence of neuropathogenesis observed in Southern Africa is due to this higher frequency of variants with dicysteine motif in Tat, we carried out functional analysis. Using both *in vitro* and mouse models, we compared the neuropathogenic properties of: (i) a Southern African HIV-1C isolate from Zambia with a dicysteine motif in Tat, HIV-1C_1084i_[33]; (ii) a Southeast Asian isolate from India, HIV-1C_IndieC1_ with a C31S substitution in Tat (iii) a HIV-1B isolate with a dicysteine motif in Tat known to cause neuronal damage and neurocognitive deficits in SCID-HIVE mouse model, HIV-1B_ADA_. We report that *in vitro*, the Southern African HIV-1C isolate, in sharp contrast to Southeast Asian HIV-1C_IndieC1_, behaved similar to HIV-1B isolates in its robust ability to recruit monocytes, induce β chemokines and cause apoptosis of human neurons. We also found that the increased monocyte recruitment and greater neurotoxicity of the Southern African HIV-1C isolate HIV-1C_1084i_ (with a dicysteine motif in Tat) is due to Tat protein using immuno-depletion. Importantly, in the SCID HIVE mouse model, HIV-1C_1084i_ caused greater neurocognitive dysfunction compared to the Southeast Asian HIV-1C_IndieC1._ Our study further corroborates the critical role that the Tat dicysteine motif plays in neuropathogenesis and suggests that it may be possible to use Tat genotyping to predict the neurocognitive outcomes of patients infected with HIV-1C.

## Results

### Geographical variation in HIV-1C Tat Exon I

**Figure 1:**
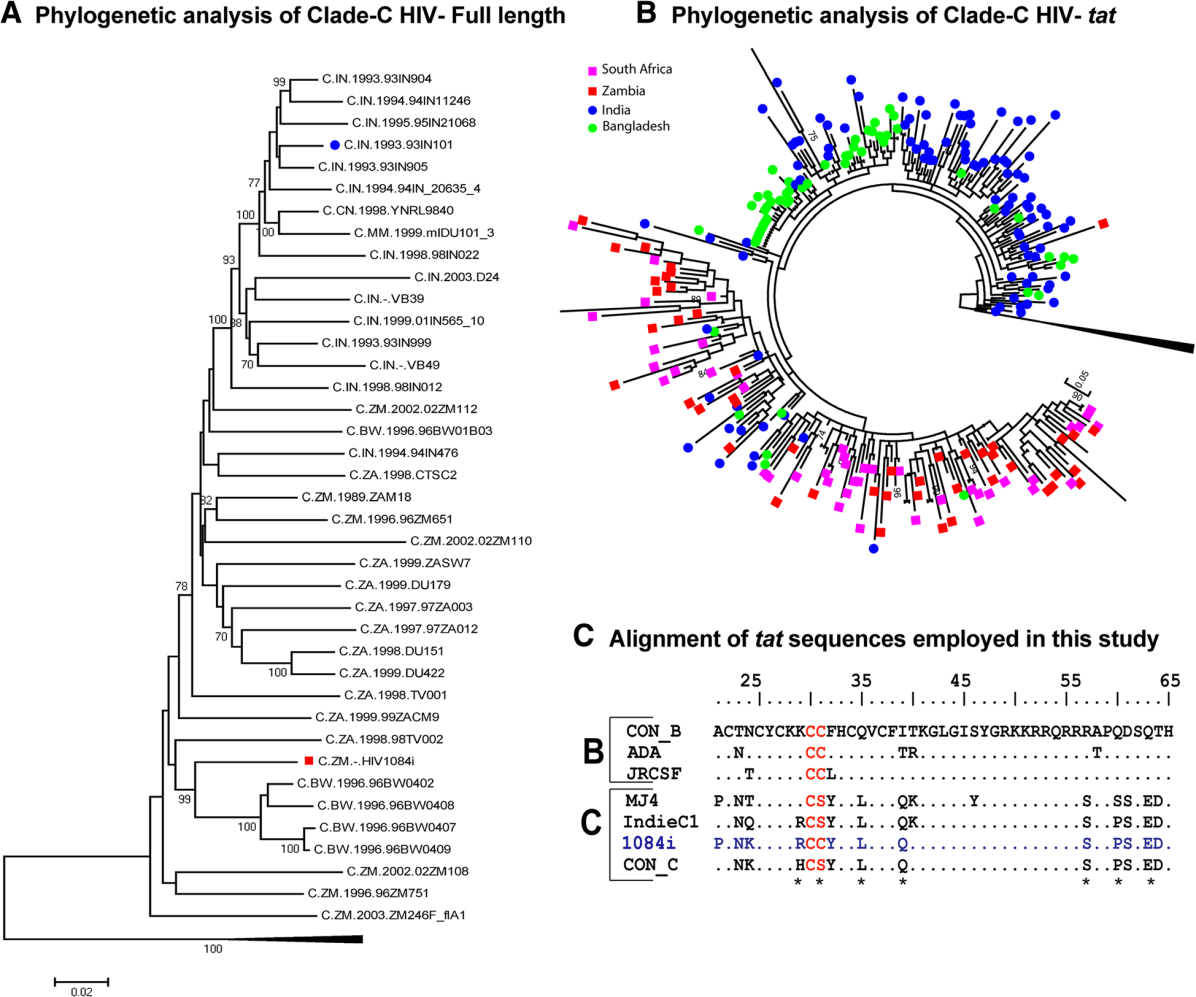
**Phylogenetic analysis using full-length clade C sequences from diverse geographical locations. A**. The graphic represents maximum likelihood phylogenetic tree using full − length clade C sequences from Southeast Asia (India, China and Myanmar) and Southern-Africa (South Africa, Zambia and Botswana) with reference clade B as out-group**.** The analysis identified a genetic divergence of HIV-1C_IndieC1_ (C.IN.93IN901 marked with filled Blue Circle) and HIV-1C_1084i_ (C.ZM.HIV1084i marked with Red square) with distinct phylogenetic clusters. **B.** Clinical isolates from two different geographical locations (Southern-Africa and Southeast Asia; *n* = 250) were included in the Maximum Likelihood (ML) phylogenetic tree along with the reference HIV-1C sequences from the Los Alamos database from Zambia, Botswana, South Africa and India (*n* =21) and HIV-1B sequences (*n* = 4) from the US, Netherlands, Thailand and France (outliers). Only the representative sequences were chosen from Los Alamos Database to avoid “cohort effect” in the phylogenetic analysis. The tree was constructed in MEGA 5 [35] software, with general time reversible with inverse gamma distribution (GTR + G + I), which has been predicted as the best-fit model. The Southern African sequences are indicated by filled squares (Red: Zambia and Pink: South Africa) and Southeast Asian sequences were marked with filled circles (Green: Bangladesh and Blue: India). **C.** The Predicted amino acid sequence of *tat* gene from the Zambian HIV-1C molecular clone HIV-1_1084i_ is aligned with sequences from representative virus isolates of HIV-1B (HIV − 1B_JR−CSF_; HIV-1B_ADA_) and HIV-1C (HIV-1C_MJ4_; HIV-1C_IndieC1_) as well as consensus sequences using ClustalW software. Clade C signature amino acid residues previously defined by Ranga et al. [18], present in the majority of HIV-1C Tat sequences in the Los Alamos database [36], are Clade C signature amino acid residues previously defined by Ranga et al. [18], present in the majority of HIV-1C Tat sequences in the Los Alamos database [36], are indicated via asterisks below consenus clade C sequence (CON_C). With the exception of C31, all these residues are conserved across all clade C isolates. Dots represent the residues identical with the consensus of the respective clade. Residues in red indicate C30C31/C30S31 motif in Tat. Sequences are grouped by clade and indicated on left.

**Figure 2:**
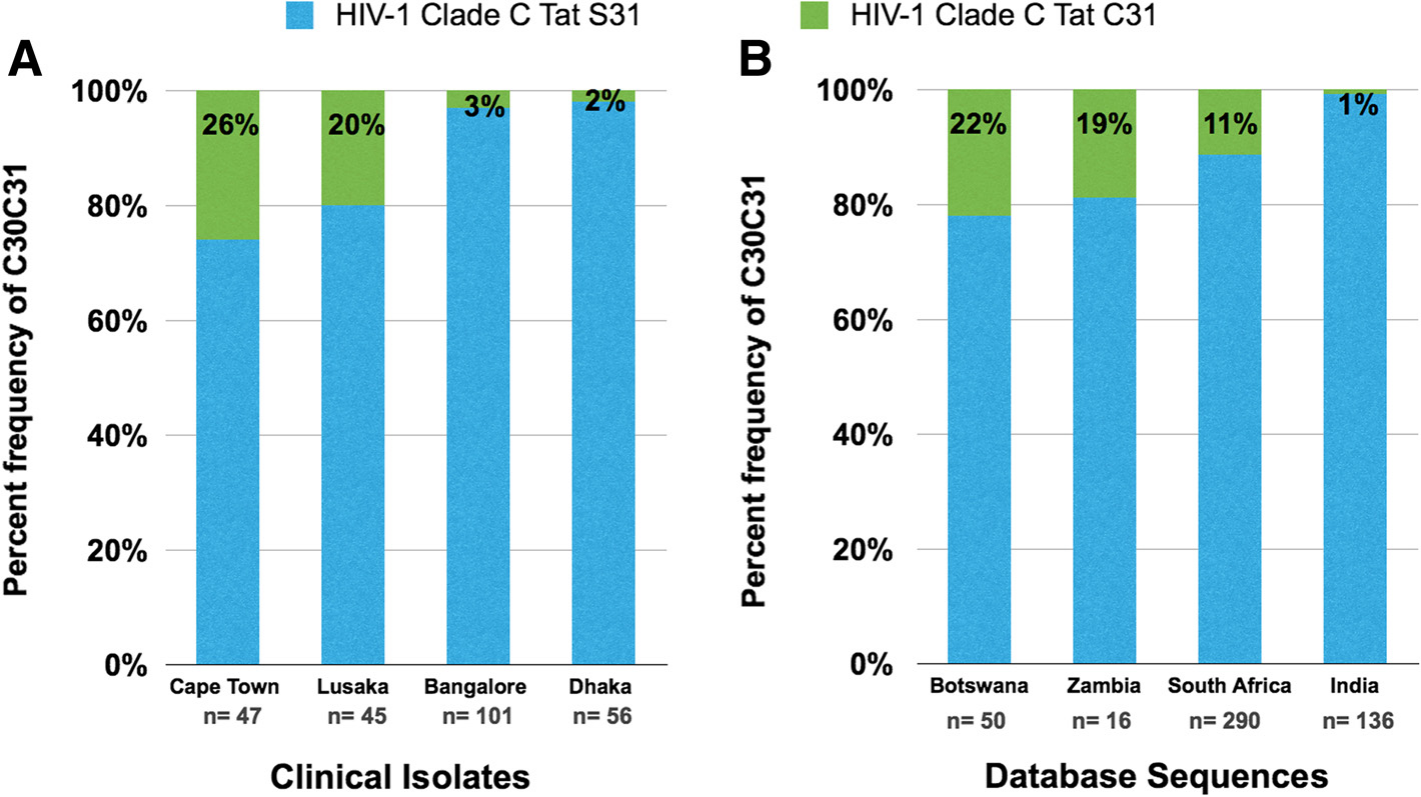
**Geographic variation in the frequency of HIV-1C Tat CC variants. A.** Frequency of HIV-1 Tat sequences with CC and CS from HIV-1-infected populations from Lusaka (Zambia), Cape Town (South Africa), Bangalore (India), and Dhaka (Bangladesh). **B.** Frequency of CC and CS variants in the sequence database among HIV-1 Tat sequences in countries where HIV-1C predominates. Sequences were downloaded from the Los Alamos database and aligned using Clustal-W software. Frequency of sequences with CC and CS at positions 30, 31 were calculated and plotted as stacked graphs. The numbers below the bars (*n*) are the total independent sequences analyzed in each case.

Based on the higher HAD prevalence rates in several Southern African countries compared to that in India, we hypothesized that the Southern African HIV-1C isolates are genetically distinct from those circulating in Southeast Asia. To test this hypothesis, we compared the full-length HIV-1C sequences derived from selected Southeast Asian (India, China and Myanmar) and Southern African (South Africa, Zambia and Botswana) countries (Ethiopian samples could not be obtained. Thus, we have limited our studies to Southern African countries). The Southern African samples were obtained from clinics in Cape Town, South Africa and Lusaka, Zambia. The Southeast Asian samples were from Bangalore, India and Dhaka, Bangladesh. Phylogenetic analysis of full-length HIV-1C sequences revealed distinct genetic differences between HIV-1C isolates from Southern African countries and those from Southeast Asia (Figure 1A). Next, we performed population sequencing of *tat* exon 1 from HIV-1 derived from clinical blood samples from two distinct geographical regions (Southern Africa & Southeast Asia) where HIV-1C is the predominant clade. Our analysis indicated that the HIV-1C Tat sequences from South Africa and Zambia formed a separate cluster segregating from the Southeast Asian HIV-1C sequences from India and Bangladesh (Figure 1B). Interestingly, the most distinguishing feature of Tat sequences from South Africa and Zambia was the relatively common occurrence of variants with an intact C30C31 motif (26% and 20% respectively), which are rare among sequences from India and Bangladesh (3% and 2% respectively; Figure 2A). Previously, when Tat sequences from worldwide were analyzed as a single group, the S31 residue emerged as a signature residue for HIV-1C Tat (frequency: 0.89) [18], while for non-clade C HIV-1 Tat, C31 was a signature residue (frequency: 0.99) [18]. In order to verify that the increased frequency of CC motif is unique to HIV-1 derived from Southern Africa, we also analyzed Los Alamos database for HIV-1 Tat sequences from South Africa, Zambia, Botswana and India. These results paralleled those obtained from analyzing the clinical isolates in that the Southern African isolates exhibit a greater frequency of occurrence of C30S31 dicystine motif (Figure 2B). This striking disparity in the proportion of variants with dicysteine motif between Southern Africa and Southeast Asia broadly reflects the HAD prevalence data from these regions [3,29,31,32,34]. Therefore, we predicted that Southern African isolates with a C30C31 motif in HIV-1 Tat are more neurovirulent. We have carried out a series of *in vitro* and *in vivo* experiments to test our prediction.

### A Southern African HIV-1C isolate with C30C31 motif

In order to test our prediction, a replication competent Southern African HIV-1C isolate retaining a dicysteine motif would be necessary. For this purpose, we exploited a Zambian HIV-1_1084i_ that was previously isolated from a pediatric patient. Previous analysis of this molecular clone showed that it was an intra-clade recombinant, where *gag* sequences clustered with Asian HIV-1C, while *pol* and *env* sequences clustered with African HIV-1C [33]. Comparison with other full-length sequences from the database confirmed that it was representative of Southern African isolates (Figure 1A). In the same analysis, we included full-length HIV-1C sequences from Southeast Asia and confirmed that the Southern African isolate (HIV-1C_1084i_) was phylogenetically different from Southeast Asian isolates (Figure 1A). Further, the ClustalW alignment of the predicted amino acid sequence of HIV-1C_1084i_*tat* exon I [33] with the consensus HIV-1B and HIV-1C sequences and other HIV-1B and C isolates (Figure 1C) revealed that this Tat sequence retains homology to other HIV-1C Tat proteins over its entire length and it was not a recombinant. However, HIV-1C_1084i_ Tat does not display the C31S polymorphism that is commonly present in HIV-1C Tat sequences. This analysis along with the fact that HIV-1C_1084i_*gag*, *pol*, and *env* sequences mapped to HIV-1C in phylogenetic analysis makes it an ideal virus to examine the neurotoxic potential of a Southern African isolate [33].

### Southern African HIV-1C_1084i_ triggers increased monocyte chemotaxis compared to Southeast Asian HIV-1C isolate

As monocyte infiltration is a hallmark of HAD, we first compared the monocyte chemotaxis properties of Southern African HIV-1C_1084i_-infected MDM supernatants with those of Southeast Asian HIV-1C and a HIV-1B isolate. In all of our studies, we have used HIV − 1C_1084i_ to represent the Southern African Tat dicysteine containing isolates, the HIV-1C_IndieC1_ as the Indian isolate with the Tat C31S variant and the US − derived, HIV-1B_ADA_ as the clade B virus prototype with Tat dicysteine. For monocyte migration and CCL2 induction, two additional virus isolates HIV-1B_JR-CSF_ (Clade B: Tat CC) [37] and HIV-1C_MJ4_ (Clade C: Tat CS) [38] were also used.

**Figure 3:**
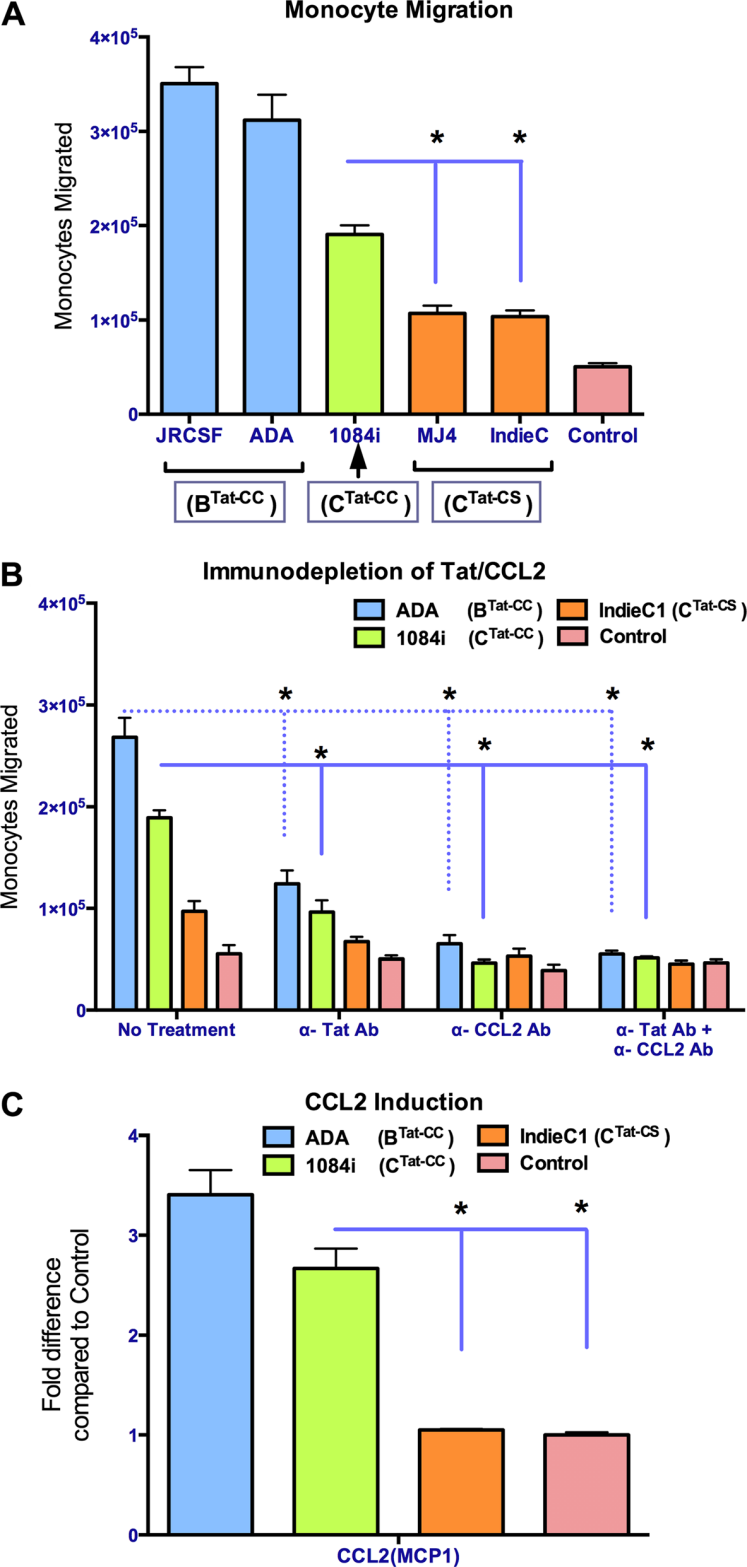
**Differential monocyte recruitment by medium from macrophages infected with different HIV-1 isolates. A.** Monocyte migration caused by medium from HIV-1 infected MDMs. At least two isolate are used for each clade. HIV-1B isolates are shown in blue, HIV-1C isolates in orange and HIV-1C_1084i_ in green. Southern African HIV-1C_1084i_ with a dicysteine motif in Tat induces significantly higher number of monocytes to migrate compared to HIV-1C IndieC1 and HIV − 1C_MJ4_. Tat genotypes of each isolate are indicated in all three panels. **B.** Determining the soluble factors in the infected MDM supernatant responsible for monocyte migration. Immuno-depletion of Tat and CCL2 resulted in significant (*) reduction of monocyte recruitment both by HIV-1_ADA_ (Clade B with dicysteine motif in Tat) and HIV-1_1084i_ (Clade C with dicysteine motif in Tat) infected supernatants. Serial immuno-depletion of both CCL2 and Tat did not further reduce background migration. **C.** Fold increases, over uninfected macrophages, in the level of CCL2 secreted by macrophages infected with various HIV-1 isolates: HIV-1B_ADA,_ HIV-1C_1084i_ and HIV-1C_IndieC1_. The levels of these chemokines in the media from uninfected control MDM cells were CCL2: 20.8ng/ml. HIV-1C_1084i_ induced significantly higher levels of CCL2 induction compared to HIV-1C_IndieC1_ and Controls. * *p* < 0.05.

The five HIV isolates indicated above were employed to infect MDMs at variable inputs to match their rates of replication, propagated for a period of 5 days and the supernatants were evaluated to ensure that similar levels of p24 (60 ng/ml − 80 ng/ml) were produced. We employed a Boyden chamber with 3μ membrane barrier and measured the ability of HIV-infected supernatants to recruit monocytes across the membrane. We found that supernatants from MDM − infected with HIV-1C_1084i_ attracts 2-fold more monocytes compared to HIV − 1C viruses lacking the dicysteine motif (HIV-1C_IndieC1_ and HIV-1C_MJ4_) (*t*(6) = 9.86, *p* < 0.0001, n = 6), but less than that observed in response to HIV-1B isolates (Figures 3A and 3B). HIV-1B isolates (HIV-1B_JRCSF_ and HIV-1B_ADA_) attracted almost 3-fold more monocytes than HIV-1C isolates lacking dicysteine motif (*t*(6) = 12.61, *p* < 0.0001, n = 6). Previously it has been established that Tat and CCL2 present in the MDM medium are responsible for increased chemotaxis caused by HIV-1B. To determine whether Tat and/or CCL2 are also responsible for increased chemotaxis induced by HIV-1_1084i_, HIV-1 Tat and CCL2 were immuno-depleted from the medium using neutralizing antibodies and monocyte migration assays were performed [39]. Immuno-depletion of Tat resulted in the reduction in recruitment of monocytes both by HIV-1B_ADA_ and HIV-1C_1084i_-infected supernatants (Figure 3B). Immuno-depletion of CCL2 also decreased the number of monocytes recruited by all three viral supernatants to control levels (Figure 3B). We note that despite immuno-depletion of Tat or CCL2, there was a background level of monocyte migration. We have used low serum (2.5%) medium for 24 h to reduce migration caused by chemotaxis factors present in the human serum. However, this basal level of migration, possibly caused by the presence of other β-chemokines in the serum, could not be reduced further even after serially immune-depleting both Tat and CCL2 (Figure 3B). Our results suggest that the increased migration of monocytes induced by HIV-1C_1084i_ is largely due to presence of Tat and CCL2 in the HIV-infected MDM medium.

We previously showed that HIV-1C_IndieC1_ induces lower levels of CCL2 chemokine than HIV-1B in infected MDMs correlating to their differences in neuropathogenesis [19,40]. We analyzed CCL2 induction for the different HIV-1 isolates studied above. Interestingly, Southern African HIV-1C_1084i_ induced 2.5 − fold greater levels of CCL2 (*t*(16) = 10.13, *p* < 0.0001) compared to uninfected controls (Control CCL2: 20.8 ng/ml) as well as when compared to HIV-1C_IndieC1_, which caused no change in the induction of CCL2 (*t*(16) = .3010, *p* > 0.5) levels from uninfected controls (Figure 3C). These results suggest that Southern African HIV-1C virus isolates with an intact dicysteine motif, similar to HIV-1B isolates, are potent inducers of chemokines, a key factor in recruitment of mononuclear phagocytes to HIV-infected sites of the brain in HAND (Figure 3C).

### Southern African HIV-1C_1084i_ Tat protein is more neurotoxic when compared to TatC from HIV-1C_IndieC1_

**Figure 4:**
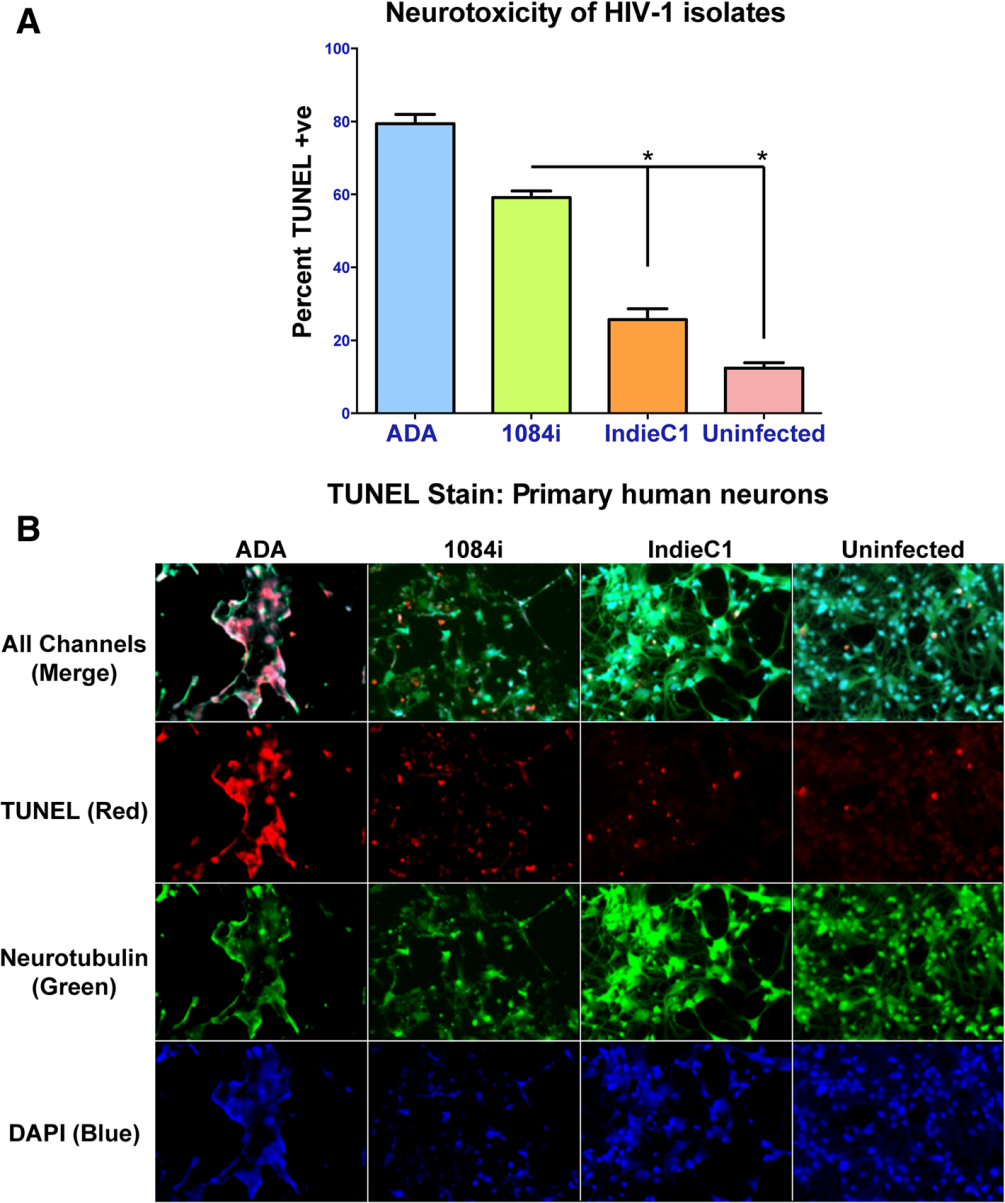
**Effect of HIV-infected MDM supernatants on neuronal apoptosis. A.** HIV-1 infected MDM and uninfected MDM supernatant were diluted with 100 μl of neurobasal medium and incubated with primary human neurons in Matek plates for 18 hours. HIV-1B_ADA_ and HIV-1C_1084i_ treatments lead to a greater loss of neuronal viability when compared to HIV-1C_IndieC1_ and uninfected supernatant. **B.** Tunnel stained primary human neurons co-stained with anti-neurotubulin antibodies and DAPI (Prolong Gold antifader with DAPI, Invitrogen). The four panels show representative fields for neurons treated with HIV-1B_ADA_, HIV-1C_1084i_, HIV-1C_IndieC1_ and media from uninfected macrophages from left to right respectively. The individual (or all) channels are indicated at left. * *p* < 0.05.

**Figure 5:**
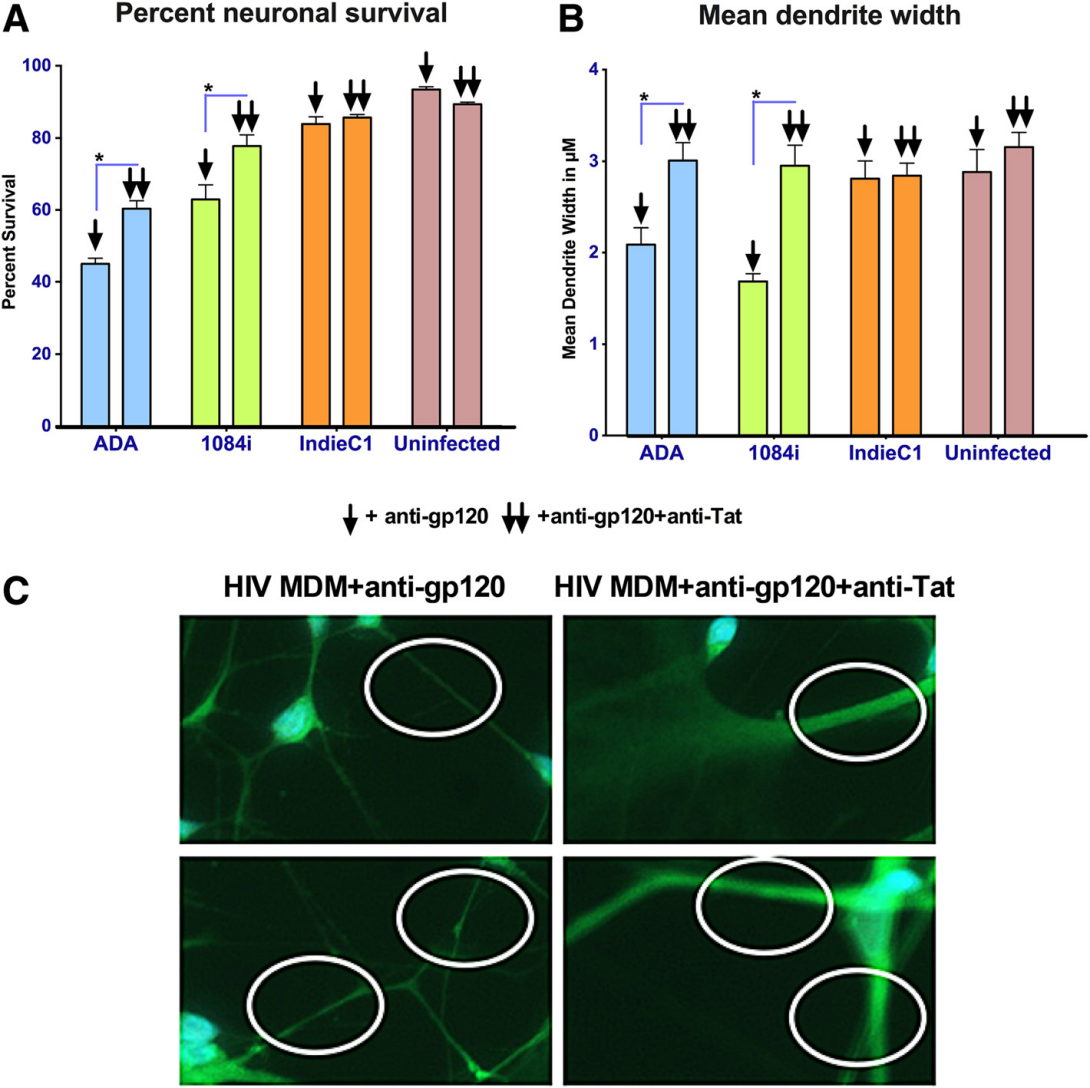
**Neurotoxicity of Tat in HIV-1C**_**1084i**_**-infected MDM supernatant. A.** HIV-infected media used in Figure 5 were used to pre-adsorb HIV particles and/or free gp120 to eliminate confounding factors and the Tat protein from such media were further adsorbed using anti-Tat antibodies to demonstrate the neurotoxicity specifically due to Tat from HIV − 1B_ADA_ and HIV-1C_1084i_ but not HIV-1C_IndieC1_. For both panels A and B, bars corresponding to medium depleted of virus/gp120 is shown by ↓, whereas those corresponding to medium depleted of both virus/gp120 and Tat protein are shown by ↓↓. **B.** Measurement of dendritic width shows HIV-1B_ADA_ & HIV-1C_1084i_ cause significant decreases in dendritic width. Use of virus-adsorbed medium and virus- plus Tat-adsorbed HIV-1B_ADA_ and HIV-1C_1084i_-infected medium as in Panel A above lead to increase in dendrite width, but not in HIV-1C_IndieC_-infected medium, clearly demonstrating the role played by HIV-1C_1084i_ Tat in neuronal atrophy. **C.** Representative fields showing effect of Tat immune-depletion in HIV-1C_1084i_ MDM media on dendrite width. The left top and bottom panels show representative fields of primary neurons treated with HIV-1C_1084i_ MDM media prior to Tat immuno-depletion and the right top and bottom panels show increase in dendrite width following immune-depletion of Tat. * *p* < 0.05.

We next examined the neurotoxicity of HIV-1C_1084i_ Tat when compared to HIV-1_IndieC1_ TatC and TatB *in vitro*. Primary human neurons were treated with conditioned medium obtained from HIV-infected MDM and neuronal viability was quantified by using TUNEL assay. Immunofluorescence was used to specifically detect neurons (via staining for neurotubulin), apoptotic cells (TUNEL staining) and nuclei (DAPI staining). The neuronal viability was reduced by 60% when HIV-1C_1084i_–infected MDM supernatants were used and greater than 80% when HIV-1B_ADA_–infected MDM supernatants were used (Figure 4A and 4B)_._ Media from uninfected Macrophages and HIV-1C_IndieC1_-infected MDMs resulted in 12% to 25% cell death respectively (Figure 4A, 4B). In order to determine the relative contribution of Tat to neurotoxicity, the MDM supernatants were first depleted of virions and free gp120 using broadly neutralizing envelope antibodies, and subsequently Tat was immunodepleted from the medium. Neurons were exposed to virus/gp120-immunoadsorbed medium with and without immunodepletion of Tat. Neuronal apoptosis was significantly reduced upon immunodepletion of Tat from HIV-infected MDM medium (Figure 5A).

Key pathologic hallmarks of HIV associated dementia are neuronal atrophy and the loss of dendritic arbor [41]. In an *in vitro* setting, dendritic density, length and thickness are a proxy indicator for signs of neuronal atrophy. We chose to examine changes in dendritic thickness in response to exposure of neurons to HIV-1 conditioned medium, to evaluate neurotoxicity. We immune-depleted Tat from virus/gp120-adsorbed media and tested the effect of both virus/gp120 − adsorbed and virus/gp120- and Tat- adsorbed media on dendritic width. Analysis of the width of the neuronal processes indicated that human neurons treated with HIV-1C_1084i_- and HIV-1B_ADA_-infected MDM supernatants depleted for both virus/gp120 and Tat proteins are significantly (HIV-1C_1084i_: p value = 0.00007; HIV-1B_ADA_: p value = 0.0071) thicker than infected MDM supernatants depleted virus/gp120 (Figure 5B, 5C). We find that the dendrite width increased to match the width of untreated neurons, implicating Tat as one of the factors responsible for neuronal atrophy observed.

### Southern African HIV-1C_1084i_ induces neurocognitive deficits in SCID mice

**Figure 6:**
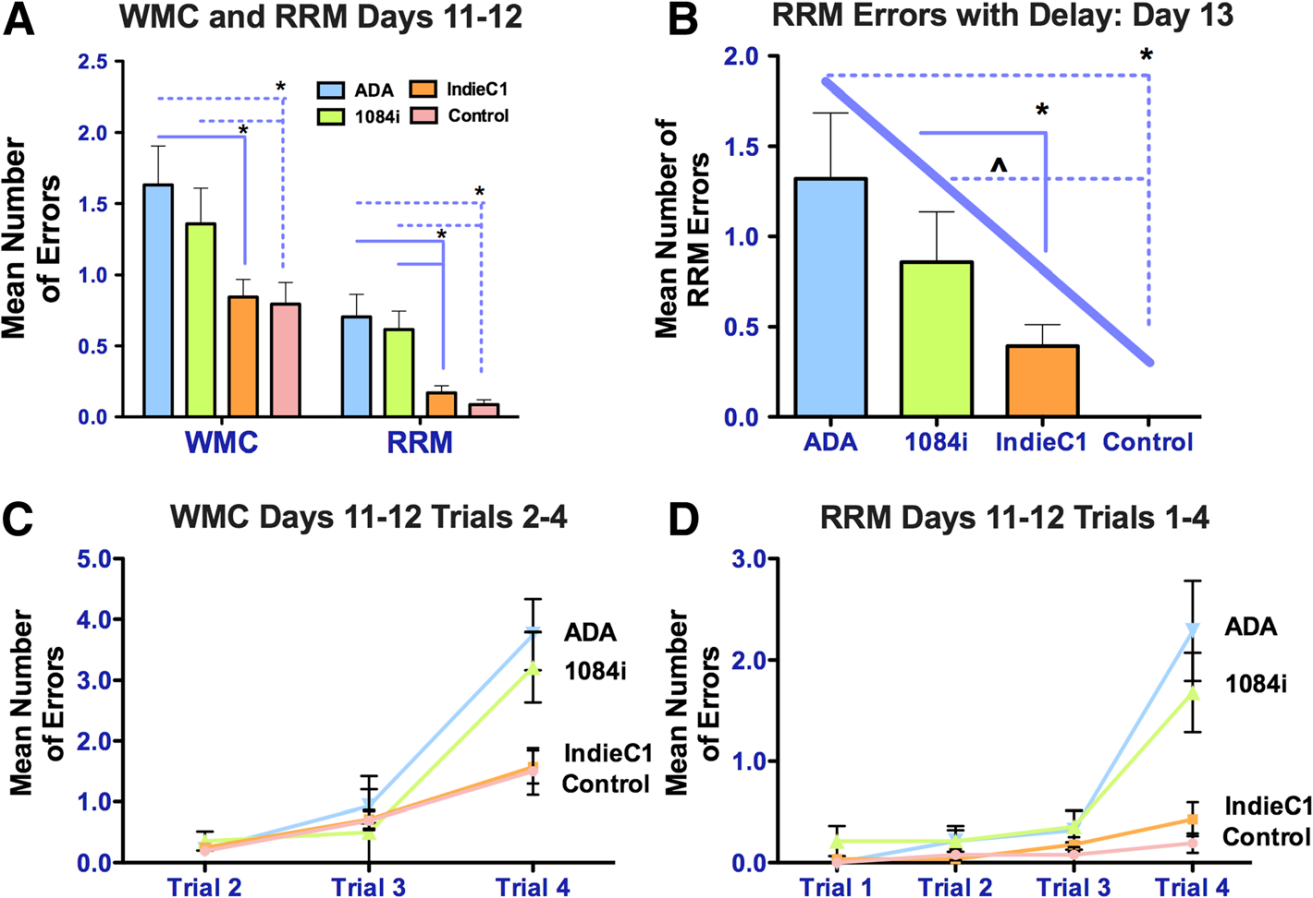
**Cognitive performance of SCID mice exposed to HIV-1 and controls evaluated on the WRAM during the asymptotic testing phase (data represent mean ±** ***SE*). A.** Analysis of working memory errors collapsed across days 11 and 12 and trials revealed Southern African HIV-1C_1084i_ and HIV-1B_ADA_ exposed mice made significantly more RRM errors than the Control (*p*s < 0.018) and HIV − 1C_IndieC1_-exposed mice **B.** RRM errors committed on Day 13 post-45m delay trials 3 and 4, the Southern African HIV-1C_1084i_ and HIV-1B_ADA_ but not HIV − 1C_IndieC1_ exposed mice committing more RRM errors as compared to the Control group. Group rankings for RRM errors committed on the delay followed a linear trend, with the Control group committing no errors, and the HIV-1B_ADA_ group committing the most. **C.** Analysis of Working Memory Correct errors committed on trial 4, when working memory load was the highest HIV-1C_IndieC1_ exposed mice did not differ from the Control group, but the Southern African HIV-1C_1084i_ and HIV-1B_ADA_ exposed mice committed more errors. * *p* < 0.05; ^ *p* < 0.05 linear trend. **D.**Evaluation of Reference Memory Repeat errors committed on trial 4, the HIV-1C_1084i_ and HIV-1B_ADA_ (i.e., mice exposed to HIV-1 with an intact dicysteine motif) committed more working memory errors when compared to the Control and HIV-1_IndieC1_ (clade C HIV-1 with C31S substitution in Tat) groups.

The SCID HIVE is a well-characterized mouse model suitable for testing HIV-induced neurovirulence and behavioral changes [20,21,42,43]. We intracranially injected male SCID mice (4 groups of mice, 6 mice/group) with human uninfected MDMs as well as MDMs infected with one of the three viruses (HIV-1B_ADA_, HIV-1C_IndieC1_ or HIV-1C_1084i_). The HIV-infected cultures were verified to contain comparable levels of virus as measured by p24 in the medium. Analysis of the mouse brain samples injected with the three different isolates showed a similar number of p24 infected macrophages (4–8 p24-positive cells per section) around the injection site. Cognitive assessment using the Water Radial Arm Maze (WRAM) comparing mice exposed to Southern African HIV − 1C_1084i_ to those exposed to Southeast Asian HIV-1C_IndieC1_ revealed the Southern African isolate affects two orthogonal measures of short term and long term working memory (working memory correct: WMC and repeat reference memory: RRM). When working memory load was highest on trial 4 during the latter most portion of testing (days 11–12 post injection), there was a significant main effect of exposure to HIV-1 isolates, HIV-1B_ADA_ and HIV-1C_1084i_ for WMC and RRM errors (WMC *F*(3,51) = 5.16, *p* = 0.003; RRM *F*(3,51) = 7.58, *p* = 0.0001; Figures 6A, 6C, &6D). The HIV-1C_IndieC1_ exposed mice did not differ from the Control group (post-hoc *p*s > 0.651; Figures 6A, 6C, &6D), whereas the HIV − 1C_1084i_ exposed mice and HIV-1B_ADA_ exposed mice committed more errors than the Control group and HIV-1C_IndieC1_-exposed mice Figures 6A, 6C, &6D). Mice exposed to HIV-1C_1084i_, in spite of being closely related to HIV-1C_IndieC1_, did not differ from mice exposed to HIV-1B_ADA_ (post-hoc *p*s > 0.238; Figures 6A, 6C, &6D).

On test day 13 post-injection, a 45m delay was imposed between trials 2 and 3 to increase the demand placed on the working memory system of the mice. There was a significant main effect for RRM on post-delay trials 3 & 4 (*F*(3,51) = 6.01, *p* = 0.0010; Figure 6B), with HIV-1C_1084i_ or HIV-1B_ADA_ (post-hoc *p*s < 0.0120), but not HIV-1C_IndieC1_, exposed mice committing more RRM errors as compared to the Control group (Figure 6B). For this measure, errors increased in a linear fashion from control animals, who committed no errors, to the HIV-1B_ADA_ exposed mice, who committed the most errors (*t*(51) = 4.27, *p* < 0.0001; Figure 6B). Our results demonstrate that exposure to Southern African HIV-1C isolate with an intact dicysteine motif in Tat causes working memory deficits similar to that caused by clade B HIV-1.

To address the strength of the relationship between each treatment group and the degree of working memory impairment, Cohen’s *d* was calculated between each group from RRM errors during test days 11–12 trials 1–4. The degree of working memory impairment, as compared to the Control group, increased from the clade C (HIV-1C_IndieC1_*; d* = 0.45) virus, to the clade C with an intact dicysteine motif (HIV-1C_1084i_*; d* = 1.08) virus and the clade B (HIV-1B_ADA_*; d* = 1.53) virus demonstrating the highest degree of working memory impairment (Figures 6A, &6C). The HIV-1C_IndieC1_ exposed mice exhibited a lesser magnitude of working memory impairment in comparison to the HIV-1C_1084i_ (*d* = 0.76) and HIV-1_ADA_ exposed mice (*d* = 1.29), and that the magnitudes of working memory impairment between the HIV-1C_1084i_ and HIV-1B_ADA_ exposed mice were similar (*d* = 0.13; Figures 6A, &6C).

## Discussion

We report that *tat* gene in the HIV-1 clade C isolates circulating in Southern African countries (South Africa, Zambia and Botswana) is evolutionarily distinct from those in Southeast Asia (India and Bangladesh). Southern African HIV-1C isolates displayed a uniquely higher proportion of variants encoding Tat protein with a dicysteine motif (C30C31) when compared to those in Southeast Asian countries. The interesting correlation between the proportion of viruses encoding Tat with a dicysteine motif and the higher prevalence of HAD in these countries [29-32] prompted us to examine the hypothesis that the presence of a dicysteine motif in clade C Tat is a determinant of neuropathogenesis. Evidence presented in this communication includes neurocognitive data in SCID-HIVE model as well as *in vitro* studies showing that a Southern African variant HIV-1C isolate (HIV − 1_1084i_) containing a dicysteine motif in Tat displays a robust ability to both recruit monocytes and to cause neurotoxicity.

Previous studies examining HIV-1 *env* sequences from Northeast India have concluded that Indian clade C has evolved into a distinct sub-clade termed C_IN_[44]. More recently, Neogi et al. [45] analyzed 168 HIV sequences drawn from 4 distinct regions of India, systematically analyzed *gag, pol* and *env* regions and concluded that the Indian clade C HIV has evolved independently from African clade C HIV over the last 40 years. They dated the origin of the Indian clade C via the most recent common ancestor (tMRCA) analysis and compared it to tMRCAs determined for clade C HIV circulating in other countries (Ethiopia, Zimbabwe and Brazil). This analysis led them to conclude that the Indian clade C probably originated from the African clade C, but has evolved independently with little intermixing with African clade C sequences [45,46]. It is generally thought that the region-specific evolution of HIV-1 clades is likely due to founder effects [44] although one cannot rule out the role of host genetic factors in perpetuating specific variants in that region [47].

The cysteine-rich domain is crucial for monocyte chemotaxis function of HIV-1B Tat protein [8] as is the case for β-chemokines [48]. The absence of dicysteine motif in HIV-1C Tat is shown to be responsible for the reduced chemotactic function [18]. Comparison of HIV-1B and HIV-1C Tat proteins showed that B Tat induces robust monocyte migration and causes the induction of chemokines, such as CCL2, while HIV-1C Tat has only a weak activity for both these properties [19]. Although it is formally possible that clade differences may be due to differential secretion or accumulation of Tat in the HIV-infected MDM medium for these two clades of HIV, we believe this to be unlikely. Li et al. [28] have previously reported that cells transfected with HIV-1C and HIV-1B Tat expression constructs secreted similar levels of Tat proteins. Furthermore, using chemically synthesized Tat proteins, Campbell et al. [22] showed that a HIV-1B Tat protein binds to CCR2, induces intracellular calcium flux, causes monocyte chemotaxis as well as induces CCL2 and TNFα, while Tat from a Southeast Asian HIV-1C (without a dicysteine motif) did not. The same group showed that Tat protein from a Southern African HIV-1C isolate (Botswanan) retaining a dicysteine motif was competent for monocyte chemotaxis and induced CCL2 and TNFα at levels similar to HIV-1B Tat protein [22]. In addition, a Southeast Asian patient isolate from Bangalore encoding a Tat protein with an intact dicysteine motif also displayed a phenotype similar to similar to Southern African HIV-1_1084i_ (Additional file 1: Figure S1).

The use of HIV-1C_1084i,_ a HIV-1C isolate from Zambia, allowed us to confirm the hypothesis that the Southern African dicysteine motif-containing HIV-1C isolates are more neuropathogenic [33]. Despite the short time span of SCID-HIVE model (4 weeks), the features exhibited in this model (gliosis, neuronal atrophy and neurocognitive deficits) are consistent with chronic encephalitis rather than an acute inflammatory state [20,21,42,43]. This model simulates the loss of memory and cognitive dysfunction observed in patients suffering from HAD/HAND and provides an effective means to compare the effects of different virus isolates [19]. The cognitive tests in mice suggest that HIV-1C_1084i_ isolate, by virtue of encoding a Tat protein with an intact dicysteine motif, can be as neurotoxic as HIV-1B viruses. Dicysteine motif of HIV-1 Tat has also been shown to be critical for direct neurotoxicity [7,28]. Thus, *in vitro* data showing direct neurotoxicity of HIV-1C_1084i_–infected MDM supernatants and SCID-HIVE data showing neurocognitive defects caused by HIV_1084i_ together support the key role played by the dicysteine motif in HIV neuropathogenesis.

Previous studies have shown that MDMs, either infected with HIV-1B [19] or exposed to purified recombinant Tat protein from HIV-1B [19,23,25], lead to a greater level of induction of inflammatory chemokines such as CCL2 than HIV-1C or Tat protein derived from it. β-chemokine levels are elevated in cerebrospinal fluid of patients with HAD [49] and HIV-1 Tat is one of the key viral proteins responsible for the induction of such chemokines [10]. Our present study showed that when an intact dicysteine motif is present in Tat (HIV-1C_1084i_), a HIV-1C virus isolate is able to induce CCL2 in sharp contrast to clade C isolates lacking the dicysteine motif (HIV-1C_IndieC1_). Our results suggest that the intact dicysteine motif is critical for the induction of inflammatory chemokines that play a crucial role in development of HAD/ HAND.

Earlier studies led to the idea that some clades of HIV-1, such as clade C, cause reduced neuropathogenesis [3,4]. Our current results highlight the fact that all clade C HIV isolates do not display a C30S31 polymorphism in the Tat protein encoded by them. Furthermore, the presence of variant clade C viruses with a C30C31 dicysteine motif can be endowed with capacity to cause neuropathogenesis. It is important to recognize that even though 99% of clade B isolates display C30C31 motif, HAD is observed in only a third of the HIV-1B-infected individuals. A broad interpretation of our current finding is that all clade C variants bearing Tat with C30C31 dicysteine motif are neurovirulent. If this notion is true, although unproven currently, it would suggest that unlike clade B, clade C virus may contain other determinants outside of *tat* gene, that when combined with the C30C31 motif, increase neurovirulence. Additional studies would be necessary to delineate the relative roles of Tat dicysteine motif and other potential neurovirulent signatures elsewhere on the Southern African subtype C isolates. One limitation of our study is that our SCID-HIVE experiments were conducted using single isolates for each of the three classes examined. This limitation will be overcome as more of well-characterized African isolates with an intact C30C31 become available. However, our conclusions are strongly supported by multiple lines of evidence from various laboratories supporting the view that the C30C31 plays multiple key roles in neuropathogenesis (monocyte infiltration, neurotoxicity and induction of proinflammatory cytokines) [8,18,19,22-25,28,50]. Based on the findings reported here, it may be possible to predict the neurocognitive outcomes for individuals newly infected with HIV-1 clade C by determining the presence of C30C31 in *tat* gene. In order to clinically validate our findings, it is necessary to conduct systematic studies in countries where HIV-1C is the dominant clade, by screening infected individuals for the presence of C30C31 versus C30S31 followed by examining their neurocognitive deficits to determine HAD.

## Conclusions

Our results demonstrate that HIV-1C isolates from Southern African countries are genetically distinct from those circulating in Southeast Asia. A greater proportion of viruses in Southern Africa display the neuropathogenesis-associated dicysteine motif in HIV Tat protein resulting in a large disparity in HIV associated neurocognitive disease in different areas of the world. Southern African isolates with HIV-1 Tat displaying dicysteine motif show more robust monocyte migration, neurotoxicity and cognitive deficits in a SCID-HIVE model than Southeast Asian HIV-1C isolates. We propose that it is possible to predict the neurocognitive outcome in millions of HIV-1C infected individuals by genotyping the HIV-1 *Tat* gene. Longitudinal HIV-1 cohort studies in Southern Africa and Southeast Asia with neuropsychiatric evaluations are required to further substantiate our conclusions.

## Methods

### Primary neurons and HIV isolates

Primary neurons were isolated and cultured as described earlier [51]. Centrifugally elutriated human monocytes were purchased from University of Nebrasaka Medical center. The monocytes were differentiated into macrophages by culturing in MCSF and 10% Human serum for a period of 7 days.

HIV-1 isolates employed for our *in vitro* and mouse model studies, HIV-1_ADA_ (US clade B), HIV-1_IndieC1_ (Indian clade C isolate) and HIV-1_1084i_ (Zambian clade C) were generated by transfection of molecular clone DNA into 293T cells and the virus released was quantitated by p24 Enzyme-linked immune-sorbent assay (ELISA; Applied Biosystems, Inc). In experiments to measure monocyte migration, two additional virus isolates HIV-1B_JR-CSF_ (Clade B: Tat CC) [37] and HIV-1C_MJ4_ (Clade C: Tat CS) [38] were also used [19,39].

### Sequence analyses, population studies and alignments

#### Study population

HIV-1-infected patient samples analyzed were from Cape Town, South Africa; Lusaka, Zambia; Bangalore, India and Dhaka, Bangladesh. In Bangalore and Dhaka, new studies were initiated while pre-existing patient samples in Cape Town and Lusaka were used to obtain *tat* sequences for our analysis. Ethical approval for this study was obtained from the Einstein Institutional Review Board (IRB) as well as from all local IRBs. See Additional file 2: Table S1 for patient demographics.

### Full length sequence analysis

Full-length HIV-1C sequences derived from Southeast Asian (India, China and Myanmar) and Southern African (South Africa, Zambia and Botswana) countries where HIV-1C is the predominant clade were obtained along with Subtype B reference sequences from the Los Alamos HIV-1 database. The sequences were aligned using Clustal-W2. Maximum likelihood phylogenetic tree was constructed based on General Time reversible model with representative sequences. Phylogenetic analyses were conducted in MEGA5 [35].

### Generating and analyzing of HIV-1 Tat sequences

For genotyping *tat*, whole blood was drawn from infected patients. Genomic DNA was isolated from either peripheral blood mononuclear cells or from whole blood and HIV-1 clade was determined based on *gag* and *env* sequencing using Maximum Likelihood (ML) phylogenetic tree in Molecular Evolutionary Genetics Analysis version 5 (MEGA5) [35]. HIV-1 *tat* exon 1 was PCR amplified from genomic DNA using primer sequences shown in Additional file 3: Table S2. Sequencing was via primers located well upstream of Exon 1 (nucleotide 5781 from HxB2 reference clone). The PCR primers were designed to amplify the entire *tat* exon 1 from the 3′ end of the *vpr* (HXB2 position 5781) up to the 5′ end of *vpu* (HXB2 terminal position 6242), with the *tat* exon 1 lying between HXB2 position 5831 and 6045). Thus, the amplicon has a region of 50 nucleotides upstream and 200 nucleotides downstream of the *tat* exon 1 sequence. All study sequences were screened for PCR cross-contamination using neighbor joining phylogenetic tree. All study sequences demonstrated well-separated branches, indicating the absence of cross-contamination. After excluding partial and problematic sequences, the percent frequency of sequences with intact dicysteine motifs was determined. The data were plotted using Graphpad Prism (v5.1, GraphPad Software, La Jolla, CA).

HIV-1 *tat* sequences that were already available prior to our study were obtained from the Los Alamos database from countries where HIV-1C was the most prevalent clade. Tat amino acid sequences were generated by translation were aligned using Clustal-W2 [52]. Sequences with premature stop codons in its open reading frame (ORF) and frame-shifts were excluded. Total numbers of HIV-1C Tat sequences with intact dicysteine motifs were enumerated and percentages derived.

### HIV-1 Infection of MDMs

Elutriated primary human monocytes (University of Nebraska Medical Center) were differentiated into macrophages and infected with HIV-1B_ADA_[53], HIV − 1C_IndieC1_[54] or with HIV-1C_1084i_[33] and propagated for a period of 14 days as described previously [19]. A comparable level of virus production was achieved among different viruses as described previously by varying the multiplicity of infection to achieve similar levels of virions in the medium following 14-day propagation. Equivalence of virus levels between the cultures was determined by p24 staining of infected MDMs [19,39] and by measuring the p24 levels in the medium.

### Monocyte migration studies and CCL2 induction

#### Monocyte migration

Human MDMs were cultured for 5 d under the same conditions as described above. Approximately 2 × 10^5^ MDMs were plated in the lower wells of a 24-well plate [19,39]. MDMs were infected with HIV-1_ADA_ (clade B) at an MOI of 0.1 for 1 h, HIV-1_Indie-C1_ (clade C with C31S substitution in Tat) at an MOI of 0.01 for 3 h and HIV-_11084i_ (clade C with dicysteine in Tat) at an MOI of 0.1 for JRCSF, and a MOI of 1 for MJ4 for 5 d. ELISA for p24 in the supernatants showed equal viral loads (60 ng/ml to 80 ng/ml p24) for all viruses. Three hundred microliters of HIV-1 infected MDM supernatant was added to the bottom chamber and 500,000 monocytes were added to the transwell. The number of monocytes migrated were analyzed after 24 hours by counting the cells in the bottom chamber using a hemocytometer. The bottom well is treated with Accutase (Millipore) prior to resuspending the cells from the bottom chamber to ensure there are no adherent monocytes that are missed.

### Tat and CCL2 immuno-depletion

Tat and CCL2 were immunodepleted from HIV-1 infected MDM supernatants using either anti-Tat (E 1.1; [50]) or anti-CCL2 antibodies (MAb 279; R&D systems) as described [19,39]. For this purpose anti-Tat and anti-CCL2 antibodies were bound to Pansorbin beads (Calbiochem, Cat no. 507861) on ice for an hour. After incubation of the antibodies with the beads (Pansorbin) the beads are centrifuged and resuspended in a small volume, 25 μl, and added to the conditioned medium obtained from the HIV-infected MDM. Following incubation for an hour, the beads are pelleted and the supernatant, without the beads, are used for the migration assay as described above.

### Measurement of CCL2

CCL2/MCP-1 levels were measured using an ELISA kit from Invitrogen (MCP-1 Human ELISA Kit- KHC 1011) in HIV-1-infected MDM supernatants collected on day 14 from five different virus isolates and from uninfected MDMs. On Day 13, MDMs were incubated for 24 h in fresh medium without human serum to eliminate the contribution of CCL2 in human serum. The day 15 no-serum supernatant was collected, and ELISA for CCL2 (R&D Systems) was performed in triplicates in three separate experiments [19].

### Neurotoxicity

Primary human neurons were isolated and cultured as described earlier [51]. Approximately 100,000 primary neurons were plated on coated MatTek well plates for a period of 2 days and allowed to stabilize and differentiate. HIV-1 infected MDM (HIV-1B_ADA_(14.8 ng/ml), HIV-1C_IndieC_ (16.2 ng/ml), HIV-1C_1084i_ (15.7 ng/ml)) & the uninfected MDM supernatant (100 μl) were diluted with 100 μl of Neurobasal medium (Gibco) and then added to primary Neurons. Following incubation for 18 hours at 37°C, neurons were fixed and TUNEL assay was performed using the TMR in situ hybridization kit (Roche; Cat No. 12156792910). Neurons were also stained using anti-neurotubulin antibodies (Abcam cat. No. 21058) and 4′,6-diamidino-2-phenylindole (DAPI; Prolong gold anti-fade agent with DAPI, Invitrogen, cat number, p36931) to distinguish them from astrocytes. Image capture and analysis was done using Nikon (NIS elements) advanced research software. Percent apoptosis/survival was determined based on the proportion of cells with DAPI stain that were TUNEL-positive. Dendrite width was measured using Nikon (NIS elements) advanced research software and median dendrite width was calculated using GraphPad Prism (v5.1, GraphPad Software, La Jolla, CA).

### SCID HIVE mouse model

#### Intracranial injection of HIV-infected MDMs

HIV-infected MDMs prepared as described above were employed for intracranial injection. At the end of a 14-day in vitro propagation of MDMs, prior to intracranial injection, the p24 levels in the supernatants of MDMs infected with HIV-1B_ADA_, HIV-1C_1084i_ and HIV-1C_IndieC1_ were 76.6 ng/ml, 63.8 ng/ml and 78.9 ng/ml respectively. Approximately 10^5^ MDMs, uninfected or infected with one of the three viruses were injected intracranially (i.c.) into 4-week old B6.CB17-*Prkdc* SCID/Szj male mice (Jackson Laboratories) as before [19,55]. Upon analyzing the mouse brain samples injected with the three different isolates, they too exhibited a similar number of HIV-infected macrophages (4–8 p24-positive cells per section) around the injection site.

### Cognitive assessment of mice

Twenty-four male mice (B6.CB17-*Prkdc* SCID/Szj) were injected with MDMs (i.e., 6 HIV-1C_1084i_, 6 HIV-1B_ADA_, 6 HIV-1C_IndieC1_ and 6 control, uninfected MDMs). Following the i.c. injection of MDMs, mice were allowed to recover for 5 days followed by testing using win-shift Water Radial Arm Maze (WRAM) as previously described [19]. The win-shift WRAM utilizes water escape onto hidden platforms as the reinforcer [19,55-57]. The testing protocol included an asymptotic phase (days 11–12) used for statistical analysis [56,58]. Working memory correct (WMC) errors were defined as entries into an arm wherein a platform had already been located, reference memory (RM) errors were an initial entry into an arm that never contained a platform, and reference memory repeat (RRM) errors were repeat entries into an arm that never contained a platform and were all quantified based on Jarrard and colleagues’ [59] orthogonal measures. On day 13, a 45 minute delay was imposed between trials 2 and 3 to assess retention of multiple items of spatial information [60]. The dependent measures for performance on the delay day were total WMC, RRM, and RM errors on trials 3 and 4, for trials after the 45 minute delay.

### Statistical analysis

Statistical analysis for the in-vitro studies was performed using Grouped Analysis and plotted in Graphpad Prism 5.1. For WRAM studies, data were analyzed using an omnibus mixed model ANOVA with Treatment as the between variable, and Days and/or Trials as the within variable(s). Follow-up comparisons employed Fisher protected least squares difference (PLSD) post-hoc tests when a significant omnibus ANOVA was found. Using SPSS (version 18, IBM, Somers, NY), polynomial contrasts were employed to assess trends in the data between treatment groups and errors committed on the WRAM. Cohen’s *d* was calculated for specific pairwise comparisons between the HIV-infected groups and the control group. Cohen’s *d* was employed as a measure of effect size that assesses the difference between two group means represented in standard deviation units [61].

## Endnote

Sequences generated for this manuscript have been deposited in Genbank. The accession numbers are: India- JQ241040-JQ241062 and JQ241064 - JQ241139; Zambia- JX661510-JX661554; Bangladesh- JX675467-JX675522; South Africa- JX675523- JX675569.

## Abbreviations

HIV-1: Human immunodeficiency virus type 1; HAND: HIV associated neurocognitive disorders; HAD: HIV associated dementia; MND: Minor neurocognitive disorders; SCID: Severe combined immune deficiency; HIVE: HIV encephalitis; WMC: Working memory errors; RRM: Repeat reference memory; MDM: Monocyte derived macrophages; ELISA: Enzyme-linked immune-sorbent assay.

## Competing interests

The authors declare that they have no competing interests.

## Authors’ contributions

VRR generated viruses and virus infected cells, conducted all in vitro tests, performed cognitive studies and drafted the manuscript; UN conducted all sequence analysis; JST carried out the statistical analysis for the cognitive testing; LP helped conduct a key aspect of cognitive test; MR generated Bangladeshi sequences; CF-F helped with the cognitive testing; SG generated the Zambian *tat* sequences; RMR generated the Zambian HIV-1C_1084i_ clone from HIV-1C 1084 viral isolates obtained by CW and SG; UR supervised the isolation of the HIV_SJ_ clone and provided the anti-Tat antibody E1.1; TA designed the cross-sectional study for Bangladesh; JJ designed the cross-sectional study for South Africa and provided the Tat sequences; EE isolated human primary neurons and provided expertise in measuring neuronal apoptosis; AS designed the cross-sectional study for India; HB-N helped design the cognitive testing and helped draft the manuscript; WRT designed the overall cognitive testing, supervised the cognitive testing and helped draft the manuscript; VRP designed the overall study, supervised all in vitro tests, conceived and initiated the cross − sectional studies, initiated and coordinated the collaborative effort and helped draft the manuscript. All authors read and approved the final manuscript.

## Supplementary Material

Additional file 1: Figure S1 A new isolate of HIV-1C^TatCC^ behaves similar to HIV-1_1084i_ in stimulating monocyte migration and CCL2 release. A. Monocyte migration caused by medium from HIV-1 infected PBMCs. Southern African HIV-1C_1084i_ and Indian HIV-1C_IN1290SJ_ with a dicysteine motif in Tat induce higher number of monocytes to migrate compared to HIV-1C IndieC1 and Control. B. Fold increases, over uninfected PBMCs (which produced 1ng/ml), in the levels of CCL2 secreted by PBMCs infected with various HIV-1 isolates: HIV-1B_ADA,_ HIV-1C_1290SJ_, HIV-1C_1084i_ and HIV-1C_IndieC1_. HIV-1C_1084i_ and HIV-1C_1290SJ_ induced higher levels of CCL2 release compared to HIV-1C_IndieC1_ and Controls. *p* values indicated in panels A and B were calculated using Fisher’s PSLD test. Click here for file

Additional file 2: Table S1 Patient demographics and clinical status. Click here for file

Additional file 3: Table S2 Tat primers used in analysis of patient samples. Click here for file

## References

[B1] Antinori A Arendt G Becker JT Brew BJ Byrd DA Cherner M Clifford DB Cinque P Epstein LG Goodkin K Updated research nosology for HIV-associated neurocognitive disorders Neurology 2007 69 1789 1799 10.1212/01.WNL.0000287431.88658.8b 17914061 PMC4472366

[B2] Geretti AM HIV-1 subtypes: epidemiology and significance for HIV management Curr Opin Infect Dis 2006 19 1 7 10.1097/01.qco.0000200293.45532.68 16374210

[B3] Satishchandra P Nalini A Gourie-Devi M Khanna N Santosh V Ravi V Desai A Chandramuki A Jayakumar PN Shankar SK Profile of neurologic disorders associated with HIV/AIDS from Bangalore, south India (1989–96) Indian J Med Res 2000 111 14 23 10793489

[B4] Clifford DB Mitike MT Mekonnen Y Zhang J Zenebe G Melaku Z Zewde A Gessesse N Wolday D Messele T Neurological evaluation of untreated human immunodeficiency virus infected adults in Ethiopia J Neurovirol 2007 13 67 72 10.1080/13550280601169837 17454450

[B5] Langford TD Letendre SL Larrea GJ Masliah E Changing patterns in the neuropathogenesis of HIV during the HAART era Brain Pathol 2003 13 195 210 12744473 10.1111/j.1750-3639.2003.tb00019.x PMC4842209

[B6] Sacktor N Lyles RH Skolasky R Kleeberger C Selnes OA Miller EN Becker JT Cohen B McArthur JC HIV-associated neurologic disease incidence changes: multicenter AIDS cohort study, 1990–1998 Neurology 2001 56 257 260 10.1212/WNL.56.2.257 11160967

[B7] Li W Galey D Mattson MP Nath A Molecular and cellular mechanisms of neuronal cell death in HIV dementia Neurotox Res 2005 8 119 134 10.1007/BF03033824 16260390

[B8] Albini A Benelli R Giunciuglio D Cai T Mariani G Ferrini S Noonan DM Identification of a novel domain of HIV tat involved in monocyte chemotaxis J Biol Chem 1998 273 15895 15900 10.1074/jbc.273.26.15895 9632634

[B9] Beall CJ Mahajan S Kuhn DE Kolattukudy PE Site-directed mutagenesis of monocyte chemoattractant protein-1 identifies two regions of the polypeptide essential for biological activity Biochem J 1996 313 Pt 2 633 640 8573103 10.1042/bj3130633 PMC1216954

[B10] Conant K Garzino-Demo A Nath A McArthur JC Halliday W Power C Gallo RC Major EO Induction of monocyte chemoattractant protein-1 in HIV-1 Tat-stimulated astrocytes and elevation in AIDS dementia Proc Natl Acad Sci USA 1998 95 3117 3121 10.1073/pnas.95.6.3117 9501225 PMC19704

[B11] Weiss JM Nath A Major EO Berman JW HIV-1 Tat induces monocyte chemoattractant protein-1-mediated monocyte transmigration across a model of the human blood–brain barrier and up-regulates CCR5 expression on human monocytes J Immunol 1999 163 2953 2959 10453044

[B12] Albini A Ferrini S Benelli R Sforzini S Giunciuglio D Aluigi MG Proudfoot AE Alouani S Wells TN Mariani G HIV-1 Tat protein mimicry of chemokines Proc Natl Acad Sci USA 1998 95 13153 13158 10.1073/pnas.95.22.13153 9789057 PMC23742

[B13] Nath A Psooy K Martin C Knudsen B Magnuson DS Haughey N Geiger JD Identification of a human immunodeficiency virus type 1 Tat epitope that is neuroexcitatory and neurotoxic J Virol 1996 70 1475 1480 8627665 10.1128/jvi.70.3.1475-1480.1996 PMC189968

[B14] Bansal AK Mactutus CF Nath A Maragos W Hauser KF Booze RM Neurotoxicity of HIV-1 proteins gp120 and Tat in the rat striatum Brain Res 2000 879 42 49 10.1016/S0006-8993(00)02725-6 11011004

[B15] Nath A Human immunodeficiency virus (HIV) proteins in neuropathogenesis of HIV dementia J Infect Dis 2002 186 Suppl 2 S193 S198 12424697 10.1086/344528

[B16] Maragos WF Tillman P Jones M Bruce-Keller AJ Roth S Bell JE Nath A Neuronal injury in hippocampus with human immunodeficiency virus transactivating protein, Tat Neuroscience 2003 117 43 53 10.1016/S0306-4522(02)00713-3 12605891

[B17] Kaul M Garden GA Lipton SA Pathways to neuronal injury and apoptosis in HIV-associated dementia Nature 2001 410 988 994 10.1038/35073667 11309629

[B18] Ranga U Shankarappa R Siddappa NB Ramakrishna L Nagendran R Mahalingam M Mahadevan A Jayasuryan N Satishchandra P Shankar SK Prasad VR Tat protein of human immunodeficiency virus type 1 subtype C strains is a defective chemokine J Virol 2004 78 2586 2590 10.1128/JVI.78.5.2586-2590.2004 14963162 PMC369202

[B19] Rao VR Sas AR Eugenin EA Siddappa NB Bimonte-Nelson H Berman JW Ranga U Tyor WR Prasad VR HIV-1 clade-specific differences in the induction of neuropathogenesis J Neurosci 2008 28 10010 10016 10.1523/JNEUROSCI.2955-08.2008 18829958 PMC2572723

[B20] Persidsky Y Limoges J McComb R Bock P Baldwin T Tyor W Patil A Nottet HS Epstein L Gelbard H Human immunodeficiency virus encephalitis in SCID mice Am J Pathol 1996 149 1027 1053 8780406 PMC1865151

[B21] Avgeropoulos N Kelley B Middaugh L Arrigo S Persidsky Y Gendelman HE Tyor WR SCID mice with HIV encephalitis develop behavioral abnormalities J Acquir Immune Defic Syndr Hum Retrovirol 1998 18 13 20 10.1097/00042560-199805010-00003 9593453

[B22] Campbell GR Watkins JD Singh KK Loret EP Spector SA Human immunodeficiency virus type 1 subtype C Tat fails to induce intracellular calcium flux and induces reduced tumor necrosis factor production from monocytes J Virol 2007 81 5919 5928 10.1128/JVI.01938-06 17376903 PMC1900281

[B23] Gandhi N Saiyed Z Thangavel S Rodriguez J Rao KV Nair MP Differential effects of HIV type 1 clade B and clade C Tat protein on expression of proinflammatory and antiinflammatory cytokines by primary monocytes AIDS Res Hum Retroviruses 2009 25 691 699 10.1089/aid.2008.0299 19621989 PMC2853861

[B24] Wong JK Campbell GR Spector SA Differential induction of interleukin-10 in monocytes by HIV-1 clade B and clade C Tat proteins J Biol Chem 2010 285 18319 18325 10.1074/jbc.M110.120840 20378550 PMC2881757

[B25] Mishra M Vetrivel S Siddappa NB Ranga U Seth P Clade-specific differences in neurotoxicity of human immunodeficiency virus-1 B and C Tat of human neurons: significance of dicysteine C30C31 motif Ann Neurol 2008 63 366 376 10.1002/ana.21292 18074388

[B26] Eugenin EA King JE Nath A Calderon TM Zukin RS Bennett MV Berman JW HIV-tat induces formation of an LRP-PSD-95- NMDAR-nNOS complex that promotes apoptosis in neurons and astrocytes Proc Natl Acad Sci USA 2007 104 3438 3443 10.1073/pnas.0611699104 17360663 PMC1805607

[B27] Liu Y Jones M Hingtgen CM Bu G Laribee N Tanzi RE Moir RD Nath A He JJ Uptake of HIV-1 tat protein mediated by low-density lipoprotein receptor-related protein disrupts the neuronal metabolic balance of the receptor ligands Nat Med 2000 6 1380 1387 10.1038/82199 11100124

[B28] Li W Huang Y Reid R Steiner J Malpica-Llanos T Darden TA Shankar SK Mahadevan A Satishchandra P Nath A NMDA receptor activation by HIV-Tat protein is clade dependent J Neurosci 2008 28 12190 12198 10.1523/JNEUROSCI.3019-08.2008 19020013 PMC6671692

[B29] Lawler K Mosepele M Ratcliffe S Seloilwe E Steele K Nthobatsang R Steenhoff A Neurocognitive impairment among HIV-positive individuals in Botswana: a pilot study J Int AIDS Soc 2010 13 15 10.1186/1758-2652-13-15 20406460 PMC2876070

[B30] Joska JA Fincham DS Stein DJ Paul RH Seedat S Clinical correlates of HIV-associated neurocognitive disorders in South Africa AIDS Behav 2010 14 371 378 10.1007/s10461-009-9538-x 19326205

[B31] Joska JA Westgarth-Taylor J Myer L Hoare J Thomas KG Combrinck M Paul RH Stein DJ Flisher AJ Characterization of HIV-Associated Neurocognitive Disorders among individuals starting antiretroviral therapy in South Africa AIDS Behav 2011 15 1197 1203 10.1007/s10461-010-9744-6 20614176

[B32] Holguin A Banda M Willen EJ Malama C Chiyenu KO Mudenda VC Wood C HIV-1 effects on neuropsychological performance in a resource-limited country, Zambia AIDS Behav 2011 15 1895 1901 10.1007/s10461-011-9988-9 21744118 PMC3314062

[B33] Grisson RD Chenine AL Yeh LY He J Wood C Bhat GJ Xu W Kankasa C Ruprecht RM Infectious molecular clone of a recently transmitted pediatric human immunodeficiency virus clade C isolate from Africa: evidence of intraclade recombination J Virol 2004 78 14066 14069 10.1128/JVI.78.24.14066-14069.2004 15564517 PMC533957

[B34] Gupta JD Satishchandra P Gopukumar K Wilkie F Waldrop-Valverde D Ellis R Ownby R Subbakrishna DK Desai A Kamat A Neuropsychological deficits in human immunodeficiency virus type 1 clade C-seropositive adults from South India J Neurovirol 2007 13 195 202 10.1080/13550280701258407 17613709

[B35] Tamura K Peterson D Peterson N Stecher G Nei M Kumar S MEGA5: molecular evolutionary genetics analysis using maximum likelihood, evolutionary distance, and maximum parsimony methods Mol Biol Evol 2011 28 2731 2739 10.1093/molbev/msr121 21546353 PMC3203626

[B36] Kuiken C Foley B Leitner T Apetrei C Hahn B Mizrachi I Mullins J Rambaut A Wolinsky S Korber B HIV sequence compendium 2010, Volume 10–03684 2010 Los Alamos, New Mexico: Theoretical Biology and Biophysics GroupNM

[B37] Koyanagi Y Miles S Mitsuyasu RT Merrill JE Vinters HV Chen IS Dual infection of the central nervous system by AIDS viruses with distinct cellular tropisms Science 1987 236 819 822 10.1126/science.3646751 3646751

[B38] Ndung’u T Renjifo B Essex M Construction and analysis of an infectious human Immunodeficiency virus type 1 subtype C molecular clone J Virol 2001 75 4964 4972 10.1128/JVI.75.11.4964-4972.2001 11333875 PMC114899

[B39] Rao VR Eugenin EA Berman JW Prasad VR Prasad VR, Kalpana GV Methods to Study Monocyte migration induced by HIV-infected cells HIV Protocols 2009 2 Springer 10.1007/978-1-59745-170-3_20 PMC4350668 19020833

[B40] Mengozzi M De Filippi C Transidico P Biswas P Cota M Ghezzi S Vicenzi E Mantovani A Sozzani S Poli G Human immunodeficiency virus replication induces monocyte chemotactic protein-1 in human macrophages and U937 promonocytic cells Blood 1999 93 1851 1857 10068657

[B41] Zheng J Thylin MR Cotter RL Lopez AL Ghorpade A Persidsky Y Xiong H Leisman GB Che MH Gendelman HE HIV-1 infected and immune competent mononuclear phagocytes induce quantitative alterations in neuronal dendritic arbor: relevance for HIV-1-associated dementia Neurotox Res 2001 3 443 459 10.1007/BF03033203 14715458

[B42] Tyor WR Power C Gendelman HE Markham RB A model of human immunodeficiency virus encephalitis in scid mice Proc Natl Acad Sci USA 1993 90 8658 8662 10.1073/pnas.90.18.8658 8378344 PMC47417

[B43] Griffin WC 3rd Middaugh LD Cook JE Tyor WR The severe combined immunodeficient (SCID) mouse model of human immunodeficiency virus encephalitis: deficits in cognitive function J Neurovirol 2004 10 109 115 10.1080/13550280490428333 15204929

[B44] Shankarappa R Chatterjee R Learn GH Neogi D Ding M Roy P Ghosh A Kingsley L Harrison L Mullins JI Gupta P Human immunodeficiency virus type 1 env sequences from Calcutta in eastern India: identification of features that distinguish subtype C sequences in India from other subtype C sequences J Virol 2001 75 10479 10487 10.1128/JVI.75.21.10479-10487.2001 11581417 PMC114623

[B45] Neogi U Bontell I Shet A De Costa A Gupta S Diwan V Laishram RS Wanchu A Ranga U Banerjea AC Sonnerborg A Molecular epidemiology of HIV-1 subtypes in India: origin and evolutionary history of the predominant subtype C PLoS One 2012 7 e39819 10.1371/journal.pone.0039819 22768132 PMC3387228

[B46] Neogi U Gupta S Sahoo PN Shet A Rao SD Ranga U Prasad VR Genetic characterization of HIV type 1 Tat exon 1 from a southern Indian clinical cohort: identification of unique epidemiological signature residues AIDS Res Hum Retroviruses 2012 28 1152 1156 22236201 10.1089/aid.2011.0380 PMC3423656

[B47] Bachu M Yalla S Asokan M Verma A Neogi U Sharma S Murali RV Mukthey AB Bhatt R Chatterjee S Multiple NF-kappaB sites in HIV-1 subtype C long terminal repeat confer superior magnitude of transcription and thereby the enhanced viral predominance J Biol Chem 2012 287 44714 44735 10.1074/jbc.M112.397158 23132857 PMC3531786

[B48] Allavena P Bianchi G Zhou D van Damme J Jilek P Sozzani S Mantovani A Induction of natural killer cell migration by monocyte chemotactic protein-1, -2 and −3 Eur J Immunol 1994 24 3233 3236 10.1002/eji.1830241249 7805752

[B49] Kelder W McArthur JC Nance-Sproson T McClernon D Griffin DE Beta-chemokines MCP-1 and RANTES are selectively increased in cerebrospinal fluid of patients with human immunodeficiency virus-associated dementia Ann Neurol 1998 44 831 835 10.1002/ana.410440521 9818943

[B50] Siddappa NB Venkatramanan M Venkatesh P Janki MV Jayasuryan N Desai A Ravi V Ranga U Transactivation and signaling functions of Tat are not correlated: biological and immunological characterization of HIV-1 subtype-C Tat protein Retrovirology 2006 3 53 10.1186/1742-4690-3-53 16916472 PMC1564039

[B51] King JE Eugenin EA Hazleton JE Morgello S Berman JW Mechanisms of HIV-tat-induced phosphorylation of N-methyl-D-aspartate receptor subunit 2A in human primary neurons: implications for neuroAIDS pathogenesis Am J Pathol 2010 176 2819 2830 10.2353/ajpath.2010.090642 20448061 PMC2877843

[B52] Larkin MA Blackshields G Brown NP Chenna R McGettigan PA McWilliam H Valentin F Wallace IM Wilm A Lopez R Clustal W and Clustal X version 2.0 Bioinformatics 2007 23 2947 2948 10.1093/bioinformatics/btm404 17846036

[B53] Theodore TS Englund G Buckler-White A Buckler CE Martin MA Peden KW Construction and characterization of a stable full-length macrophage-tropic HIV type 1 molecular clone that directs the production of high titers of progeny virions AIDS Res Hum Retroviruses 1996 12 191 194 10.1089/aid.1996.12.191 8835195

[B54] Mochizuki N Otsuka N Matsuo K Shiino T Kojima A Kurata T Sakai K Yamamoto N Isomura S Dhole TN An infectious DNA clone of HIV type 1 subtype C AIDS Res Hum Retroviruses 1999 15 1321 1324 10.1089/088922299310223 10505681

[B55] Sas AR Bimonte-Nelson H Smothers CT Woodward J Tyor WR Interferon-alpha causes neuronal dysfunction in encephalitis J Neurosci 2009 29 3948 3955 10.1523/JNEUROSCI.5595-08.2009 19321791 PMC3598587

[B56] Hyde LA Hoplight BJ Denenberg VH Water version of the radial-arm maze: learning in three inbred strains of mice Brain Res 1998 785 236 244 10.1016/S0006-8993(97)01417-0 9518631

[B57] Bimonte-Nelson HA Hunter CL Nelson ME Granholm A-CE Frontal cortex BDNF levels correlate with working memory in an animal model of Down syndrome Behav Brain Res 2003 139 47 57 10.1016/S0166-4328(02)00082-7 12642175

[B58] Bimonte HA Denenberg VH Estradiol facilitates performance as working memory load increases Psychoneuroendocrinology 1999 24 161 173 10.1016/S0306-4530(98)00068-7 10101725

[B59] Jarrard LE On the role of the hippocampus in learning and memory in the rat Behav Neural Biol 1993 60 9 26 10.1016/0163-1047(93)90664-4 8216164

[B60] Luine V Rodriguez M Effects of estradiol on radial arm maze performance of young and aged rats Behav Neural Biol 1994 62 230 236 10.1016/S0163-1047(05)80021-4 7857245

[B61] Cohen J Statistical power analysis for the behavioral sciences 1988 2 Hillsdale, N.J.: L. Erlbaum Associates

