# Supplementary figures and images for: Clade C HIV-1 isolates circulating in Southern Africa exhibit a greater frequency of dicysteine motif-containing Tat variants than those in Southeast Asia and cause increased neurovirulence
Source: Retrovirology. 2013 Jun 8;10:61. doi: 10.1186/1742-4690-10-61 (PMC3686704; doi:10.1186/1742-4690-10-61)

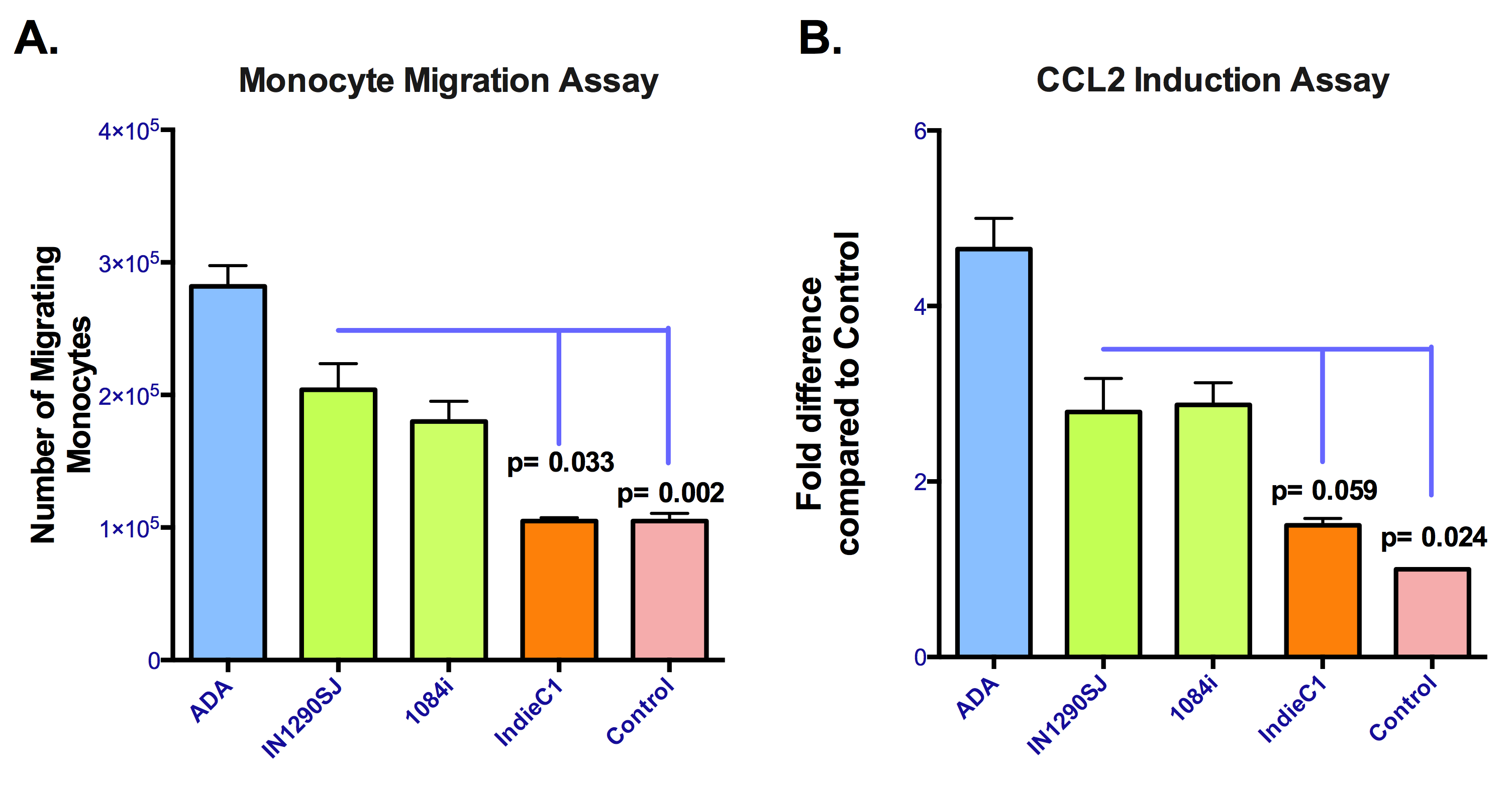

Supplement: Additional file 1: Figure S1 — A new isolate of HIV-1CTatCC behaves similar to HIV-11084i in stimulating monocyte migration and CCL2 release. A. Monocyte migration caused by medium from HIV-1 infected PBMCs. Southern African HIV-1C1084i and Indian HIV-1CIN1290SJ with a dicysteine motif in Tat induce higher number of monocytes to migrate compared to HIV-1C IndieC1 and Control. B. Fold increases, over uninfected PBMCs (which produced 1ng/ml), in the levels of CCL2 secreted by PBMCs infected with various HIV-1 isolates: HIV-1BADA, HIV-1C1290SJ, HIV-1C1084i and HIV-1CIndieC1. HIV-1C1084i and HIV-1C1290SJ induced higher levels of CCL2 release compared to HIV-1CIndieC1 and Controls. p values indicated in panels A and B were calculated using Fisher’s PSLD test. [file 1742-4690-10-61-S1.tiff]
