# Supplementary material for: Clade C HIV-1 isolates circulating in Southern Africa exhibit a greater frequency of dicysteine motif-containing Tat variants than those in Southeast Asia and cause increased neurovirulence
Source: Retrovirology. 2013 Jun 8;10:61. doi: 10.1186/1742-4690-10-61 (PMC3686704; doi:10.1186/1742-4690-10-61)
Supplement: Additional file 2: Table S1 — Patient demographics and clinical status. [file 1742-4690-10-61-S2.docx]

**Table S1: Patient Demographics and Clinical Status**

|  | **Cape Town**  **(South Africa)**  **n = 47** | **Lusaka**  **(Zambia)**  **n= 45** | **Bangalore**  **(India)**  **N = 101** | **Dhaka**  **(Bangladesh)**  **n = 56** |
| --- | --- | --- | --- | --- |
| **Gender**  **Male**  **Female** | 12 (24 %)  35 (76 %) | 9 (20%)  36 (80%) | 61 (60.1 %)  40 (39.9 %) | 49 (87.5 %)  07 (12.5 %) |
| **Age**  **Years, Mean ± SD** | 30 ± 3.1 | Not Available | 38 ± 8 | 33 ± 9 |
| **Subject Recruitment**  **Start Date**  **End Date** | February 2009  June 2009 | January 1998  December 2002 | November 2009  March 2011 | January 2003  July 2009 |
| **Clinical Status**  **Viral Load, Log copies/mL,**  **Mean (SD)**  **CD4 Count, Cells/mm^3^, Mean** ± **SD** | Not Available  209 ± 145 | Not Available  Not Available | 5.56 ± 0.06    266 ± 195 | Not Available  465 ± 210 |
| **HAART Status**  **HAART Naive**  **On HAART**  **Unknown** | 50 (100 %)  0  0 | 9 (20%)  0  36 (80%) | 87 (85.3 %)  15 (14.7 %)  0 | 29 (51.7 %)  0  27 (41.3 %) |
| **Route of Infection**  **Heterosexual**  **Homosexual**  **IDUs** | Unknown  Unknown  Unknown | 5 (11%)  Unknown  Unknown | 100 (98%)  0  0 | 22 (39.2 %)  0  29 (51.7 %) |
