# Supplementary material for: Clade C HIV-1 isolates circulating in Southern Africa exhibit a greater frequency of dicysteine motif-containing Tat variants than those in Southeast Asia and cause increased neurovirulence
Source: Retrovirology. 2013 Jun 8;10:61. doi: 10.1186/1742-4690-10-61 (PMC3686704; doi:10.1186/1742-4690-10-61)
Supplement: Additional file 3: Table S2 — Tat primers used in analysis of patient samples. [file 1742-4690-10-61-S3.docx]

|  | **HIV-1 Tat Exon 1 Primers** |
| --- | --- |
| **Bangalore, India &**  **Dhaka,**  **Bangladesh** | **F:**5’TAGTAGAGGATAGATGGAACAAGSCCCCAG3’  **R:**5’TCTGTGGGTACACAGGCATGTGTRGCCCA3’ |
| **Lusaka,**  **Zambia** | **F:**5’TAGTAGARGATAGATGGAACAAGCCCCCAG3’  **R:**5’TCTGTGGGTACACAGGCATGTGTRGCCCA3’ |
| **Cape Town,**  **South Africa** | **F:5’**GCATTCCCTACAATCCCCAAAG3’  **R:5’**CACTTCTCCAATTGTCCCTCA3’ |

**Table S2: Tat primers used in analysis of patient samples**
